# An Empirical Investigation of Virtual Networking Sites Discontinuance Intention: Stimuli Organism Response-Based Implication of User Negative Disconfirmation

**DOI:** 10.3389/fpsyg.2022.862568

**Published:** 2022-05-06

**Authors:** Weigang Ma, Anum Tariq, Muhammad Wasim Ali, Muhammad Asim Nawaz, Xingqi Wang

**Affiliations:** ^1^College of Economy and Management, Shihezi University, Shihezi, China; ^2^Institute of Agricultural Modernization, Shihezi University, Shihezi, China; ^3^School of Economics & Management, Beijing University of Posts and Telecommunications, Beijing, China; ^4^Department of Business Administration, GC Women University, Faisalabad, Pakistan; ^5^Lyallpur Business School, Government College University, Faisalabad, Pakistan; ^6^School of Management, University of Science and Technology of China, Hefei, China

**Keywords:** information disconfirmation, hedonic disconfirmation, discontinuance intention, China, virtual networking sites

## Abstract

The study’s prime objective is to investigate the user discontinuance intention in the shed of the negative disconfirmation of user expectation. The study has derived the theoretical structure from the expectancy disconfirmation theory (EDT) enacted through the stimuli organism response (SOR) framework to study the actual cause and effect relationship of human behavioral response. To investigate the user discontinuance intention behavioral response, a total of 434 correct and complete answers were shortlisted for analysis. To examine the data set, the study has used the modern partial least square method technique or simply SmartPLS service package to run the structural equation modeling (SEM). Moreover, the study has implied the 80/20 rule run the mediating analysis of the SOR framework. The statistical results show that all three stimuli make significant positive disconfirmation of the user beliefs in terms of dissatisfaction and the anxiety that ultimately leads to the discontinuance intention in virtual network users. Further, these results are validated through the six mediating relationships, which partially mediate the relationship between the stimuli and response. Besides all these findings, this study has made some practical and realistic theoretical and practical implications for both researchers and service-providing managers.

## Introduction

In the era of virtual networking sites (VNSs), such sites have gained rapid public acceptance and user audience in recent years due to the wide availability of the internet and its related application (Maier et al., 2015; Han et al., 2017). These sites include Facebook, Twitter, Instagram, and many others. Despite the repeated growth in recent times, these platforms face diverse challenges (Luqman et al., 2017; Nawaz et al., 2018). Facebook is the industry leader in terms of the number of users (Al Heeti, 2019), but they are losing their active userbase, and this challenge is growing with time (Al Heeti, 2019). Facebook is not the only one to lose this active participant, other prominent networks such as the Chinese Qzone also experienced a similar decline, and Orkut is now defunct (Smith, 2013). Considering this scenario, all the major industry players face this challenge and suffer from consumer loss of interest in VNSs, costing the businesses billions of dollars (Phu and Gow, 2018). All the major networks are struggling to build consumer interest for an extended period and enhance the maturity period of VNSs in service life cycle stages (Turel, 2014). The networks face intense challenges and are exploring the different corridors to improve services and sustain users for a longer time.

Service providers are working day and night to improve the system features to motivate the continued use of VNSs by offering new games, activities, features, and communication platforms. The recent survey findings elaborate the situation that some 61% of the users have stopped or are considering stopping using virtual networks, almost 20% have decided to leave the VNSs permanently due to boredom, gossip, bullying, abuse, envy and many other issues causing hurdles in continued use (Duggan, 2017). These facts drive more interest in user discontinuous behavior while using the VNSs. The discontinuance intention has diversified determinants than continuous intention (Dhir and Midha, 2014). The researchers have started investigating the factors that might cause this boredom and break-taking habits (Wang et al., 2011). The work of Maier et al. (2014) discussed the “discontinuance of usage” as a method to counter the stressful situation caused by VNSs because of excessive information and social activities (Sthapit et al., 2019) found social overload as one of the most prominent causes of discontinuous intention (Luqman et al., 2017) extended the concept while introducing VNS exhaustion as a possible determinant of discontinuous intention. The literature draws attention to the negative perspective of the VNSs and unwanted excessive usage leading to the pressure situation that ultimately leads to discontinuous intentions. Despite these findings, many questions need to be addressed (Divine et al., 2019).

This study investigates the post-consumption behavioral intention of VNS users. By determining the role of the disconfirmation in the context of informational disconfirmation, social disconfirmation, and hedonic disconfirmation. Further insight was required into how users experience anxiety and dissatisfaction with the VNSs platform. In this context, the current study conceptualizes the theoretical existence of the dissatisfaction and anxiety that influences the user continuation intention. The study framework is established with the help of expectancy disconfirmation theory (EDT). This study incorporates social disconfirmation, informational disconfirmation, and hedonic disconfirmation from the EDT. It adopts anxiety and dissatisfaction in mediating the EDT factors and user discontinuous intention. This integration of different perspectives brings out a robust theoretical structure that will further enhance the understanding of user discontinuous intention. Moreover, this structure will extend the current boundaries of literature. This study will identify the disconfirmation factors and their ability to influence users’ internal state while incorporating the determinants from EDT and adopting the stimuli organism response (SOR) framework.

## Literature Review

Maier et al. (2015) define the phenomena studied as “a social network site is a web-based service allowing individuals to construct a profile, articulate the connections they share with other users and view connections made by others within this network.” The most common communication methods between the users are messengers, status, blocks, emails, and many other facilities provided by VNS platforms (Lin et al., 2020). VNS platforms provide dynamic communication experiences to users with diverse psychological and sociological consequences (Xie and Tsai, 2021). These are the reasons for social scientists’ interest in VNSs and related discontinuance intentions (Wang et al., 2020).

### Expectancy Disconfirmation Theory

Expectancy disconfirmation theory is a vital consumer behavioral theory that gained a lot of appreciation from researchers to investigate the consumer dis/satisfaction intention and allied behavioral perspective (Oliver, 1980; Cai and Chi, 2021) conceptualized the theory that consumers repurchase, reuse or discontinue the customer satisfaction level with experience primarily develops the service intentions. Oliver’s classic model postulates that disconfirmation has the most immediate impact on consumer satisfaction. EDT states that consumer expectations are going to be positive or negative disconfirmed (Abrate et al., 2021). If expectations are positively disconfirmed, continuation intention would be higher, and in case of negative disconfirmation, higher the probability of discontinuation. Literature provides ample evidence that VNSs based expectations are negatively disconfirmed (Maier et al., 2014; Luqman et al., 2017; Nawaz et al., 2018). In this context, the study offers three literature enacted categories of negative disconfirmation of VNSs consumers. Namely, social disconfirmation, information disconfirmation, and hedonic disconfirmation. In the context of EDT, disconfirmation influences consumers’ inner psychological state, causing multiple service-related behavioral issues.

### Stimuli Organism Response Paradigm

The framework is adopted from the related field of psychology used to measure the mental state of respondents through three interlinked phenomena such as external environmental stimuli (R), internal mental state of the users (O), and the ultimate response of the user that is behavioral response (R). This framework has received broad appreciation from the existing body of literature (Kim et al., 2020) used the framework to investigate the influence of external environmental factors such as service delivery failure and its adverse effect on the internal state of the users influencing the repeat visit intention (Chandra and Cassandra, 2019) extend the framework into the online food delivery service sector. The study considers the external factors causing delays in food delivery, leading to the consumer’s negative cognitive feeling, eventually negatively influencing the extent of the consumers’ repeat purchases (Zhai et al., 2020)’s study considers collaborative learning as an external factor and its ability to control the learning capacity of the individual student. This internal state eventually leads to the users’ increased learning capacity and overall wellbeing. In short, the framework is validated across the social sciences studies, and the authors consider the SOR framework for VNSs behavioral outcome.

### Underpinning the Disconfirmation as Stimuli

Stimulus is the “environmental factor encountered by the individuals” (Jacoby, 1978). VNSs have interactive elements such as communication, entertainment, and socialization. The theory of traditional use and gratification “U and G” has identified three categories that meet user gratification needs: socialization, information availability, and hedonic concerns (Maier et al., 2012, 2015; Müller et al., 2017; Tugrul, 2017; Nawaz et al., 2018). The recent literature provides ample evidence supporting these factors (Ayyagari et al., 2011; Maier et al., 2014; Luqman et al., 2017; Cao and Sun, 2018). Further, virtual networks support free interaction, information flow, and gaming, encouraging web interaction and associated service consumption (Elhai et al., 2016). As this study is focused on dissatisfaction and post-consumption anxiety, our analysis considers these three perspectives of socialization, information, and hedonic as proposed from the previous literature (Ali-hassan et al., 2015).

The socialization perspective of VNS allows the users to establish limitless social links and contact, which ultimately surpasses the minimum barrier causing negative disconfirmation (Luqman et al., 2017). The information perspective allows users to view, read, and develop all kinds of information from their and peers’ walls without any restriction. When this limit is surpassed, consumer experiences negative information disconfirmation (Liao et al., 2011). The hedonic perspective allows multiple online gaming, video sharing, and conferencing options, providing free will to spend ample time on internet-based virtual networks. Literature shows that excessive engagement is causing technostress, VNS exhaustion, regret, and envy (Maier et al., 2014, 2015; Nawaz et al., 2018). These factors cause negative hedonic disconfirmation in the context of VNS.

### Underpinning the Dissatisfaction and Anxiety as Organism

The “O” is the internal state of the user. The psychological mindset of the user after experiencing the adverse perspective of the service-providing platform (Kalsnes and Larsson, 2018). The current study considers dissatisfaction and anxiety as the organism factors developed in response to the environmental stimuli as social disconfirmation, cognitive disconfirmation, and hedonic disconfirmation. The power of the organism determines the behavioral intensity. The higher the intensity of the organism, the higher the probability of a desired behavioral outcome (Kim et al., 2020).

Expectancy disconfirmation theory provides a compound solution of satisfaction and dissatisfaction (Oliver, 1977). Positive disconfirmation of beliefs leads to happiness, and negative disconfirmation causes dissatisfaction (Oliver, 1980). This study considers the post-consumption behavior of the VNSs users. The study finds the negative perspective of disconfirmation, so the study takes up dissatisfaction as the internal organism. The second perspective of the organism is anxiety. Commonly known as the emotional response to external stimuli. Anxiety is the feeling of depressive emotions such as the fearfulness of future events that might or might not occur in the future (Kim et al., 2018). Anxiety is the experiencing of stress induced by the situational context, and the individual experiencing the anxiety tries to avoid such situations (Utz and Maaß, 2018).

### Under Pinning the Discontinuance Intention as Response

The response (R) is the byproduct of the organism and can develop a positive or negative behavioral reaction (Nawaz et al., 2018). The organism causes internal instability, and the intensity of this insatiability will determine the behavioral response. The study of Um and Harrison (1998) confirms that the user’s behavioral response is mediated by emotional reactions such as dissatisfaction and anxiety. In short, the answer is motivated and is developed based on experience. Similarly, the discontinuance intention is not the sudden response of the user. Instead, it is formed over the user experiences the VNSs (Bij de Vaate et al., 2018). The disconfirmation at the informational, social, and hedonic levels leads to negative consequences such as discontinuing intention. This can be summed as the behavioral response or the result that empowers the VNSs users to consider a temporary or permanent break (Koeske and Koeske, 1993). This study observes that the extreme consequence of discontinuance intention can be the permanent distancing from the VNSs (Luqman et al., 2017).

## Hypothesis Development

The section presents the hypothesis and study structure.

### Information Disconfirmation and Anxiety and Dissatisfaction

The information a user seeks in VNSs is not one-way traffic. The user generates, shares, and sees the other’s information in pictures, status, opinions, ideas, discussions, and much more (Raacke and Bonds-Raacke, 2008; Resce and Maynard, 2018). The disconfirmation is caused by the excessive information being passed on to the end-user surpassing maximum capacity to process information leading to the information disconfirmation (Zhang et al., 2016). Excessive cognitive engagement leads to inevitable psychological and social consequences. First, the user develops a sense of dependency and addiction regarding VNS users (Flayelle et al., 2019). Second, such an excess of fallow information produces emotional disturbance that can easily influence work-family balance and lead to conflicts (Shen et al., 2015). Third, such excessive fallow information can also lead to procrastination and wasting the user’s valuable time. The user stays in touch with the social networking sites during family, friend, and work time, which takes away the precious time for personal satisfaction and growth (Fernie et al., 2016). Forth, this huge wasteland of information causes information disconfirmation that leads to emotional instability (Sicilia and Ruiz, 2010). Developing anxiety is one of the undesired outcomes of excessive engagement with the VNSs. And the anxiety leads to an adverse effect. Based on the above-stated fact, the study processes the following set of hypotheses:

**H1a. Information disconfirmation positively induces anxiety in virtual network users.**
**H1b. Information disconfirmation positively induces dissatisfaction in virtual network users.**

### Social Disconfirmation, Dissatisfaction, and Anxiety

The study of Nawaz et al. (2018) found that a single user can handle a maximum number of 124 VNSs friends. The data shows that leading social networking sites have surpassed this maximum limit and have hundreds of people in their social media accounts to respond to and share information. This demand leads to social overload, and users start to avoid such stressful situations and stay in their comfort zone (Zhang et al., 2016). This overload or stress negatively disconfirms user expectations from the VNSs and leads to multiple psychological and sociological issues. One of the prime mental disorders experienced by information technology users is anxiety. Anxiety is felt as depression, weakness, loss of control, and fear (Phillips et al., 2014). The prior literature has found the relationship between the disconfirmation and the negative consequences experienced by the technology users (Liao et al., 2007; Oh et al., 2018)’s study observed that the disconfirmation shared by high-tech technologies leads to negative health consequences. The study further proposes that:

**H2a. Social disconfirmation positively induces anxiety in virtual network users.**
**H2b. Social disconfirmation positively induces dissatisfaction in virtual network users.**

### Hedonic Disconfirmation, Anxiety, and Dissatisfaction

The VNS users have the freedom to play virtual online games, share videos, funny pictures, and much more for the sake of entertainment that keeps them engaged for a more extended period (Kowal et al., 2018). The literature shows that the maladaptive engagement with online games, videos, and content is causing many psychological issues known as hedonic disconfirmation (Maier et al., 2015). Nawaz et al. (2018) found that excessive hedonic engagement causes negative disconfirmation of the networking sites, leading to the avoidance of such networks as a coping strategy (Ohannessian, 2018) saw educational performance anxiety in online hedonic excessive users of gratification. Similarly, the study of Wang et al. (2019) finds a close relationship between the anxiety feeling and losing online gaming facilities’ user satisfaction (Basabas and Sibley, 2020) examined the relationship between online hedonic activities and user life satisfaction, self-esteem, and body anxiety. His prime findings were that excessive hedonic engagement leads to multiple psychological issues. Based on these studies, authors claim that excessive hedonic engagement with VNSs induces dissatisfaction with service and leads to an adverse emotional experience of anxiety. So based on these facts, the study proposes that:

**H3a. Hedonic disconfirmation positively induces anxiety in virtual network users.**
**H3b. Hedonic disconfirmation positively induces dissatisfaction in virtual network users.**

### Anxiety, Dissatisfaction, and Discontinuance Intention

The dissatisfaction is caused by the disconfirmation of user beliefs and expectations of the product or service. Dissatisfaction is a negative emotional feeling incurred due to the user expectations’ negative disconfirmation (Zehrer et al., 2011). This study considers the VNSs disconfirming perspective, such as the user expectation’s social, informational, and hedonic disconfirmation. These three perspectives show that when users experience disconfirmation of the platform’s core services leads to a negative feeling, causing a negative outcome for the service providers and negatively influencing the user base (Nawaz et al., 2018). The negative disconfirmation causes the dissatisfaction but the prior literature hints that it’s not only the dissatisfaction; the process leads to multiple consequences (Chiu et al., 2011). This study proposes another variable: anxiety caused by the negative disconfirmation of user expectations of the VNSs. Anxiety is the strong negative emotional feeling due to the negative disconfirmation of the VNS expectations (Oberst et al., 2017). The literature shows that when consumers experience anxiety, they are psychologically pushed to take corrective measures such as leaving or taking a break from such experiences that lead to anxiety and discomfort (Elhai et al., 2018). Therefore the study proposes that:

**H4: Dissatisfaction positively influences the user discontinuance intentions.**
**H5: Anxiety positively influences the user discontinuance intentions.**

### Mediating Relationships

**FIGURE 1:**
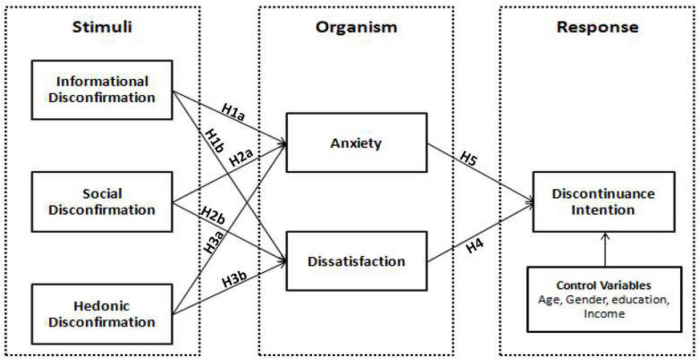
The diagram presents the framework based on the SOR paradigm.

The current context studies the organism as the byproduct of the stimuli and the organism’s behavioral response. This framework is a perfect fit to check the user experiencing the negative outcome of the excessive usage of VNSs. The literature shows that VNS users are experiencing a damaging wave of engagement. The VNSs are losing their active base of users, causing a lot of trouble to the service providers and service users (Lo, 2019). So we can claim that the organism mediates the relationship between the stimuli and behavioral response. The conceptual view of the proposed hypotheses is shown in Figure 1. So, we hypothesize that:

**H1c: Dissatisfaction mediates the relationship between informational disconfirmation and user discontinuance intentions.**
**H2d: Anxiety mediates the relationship between social disconfirmation and user discontinuance intentions.**
**H3c: Dissatisfaction mediates the relationship between Hedonic disconfirmation and user discontinuance intentions.**
**H3d: Anxiety mediates the relationship between Hedonic disconfirmation and user discontinuance intentions.**

## Methodology

This section explains the measurements employed, the sample observed for the study, and the procedure adopted for data collection.

### Measurements

To ensure the construct validity and reliability current study has adapted the measurement scales from the existing body of literature. Table 1 presents all the measurement scale items. The construct items for the information disconfirmation were adapted from the study of Maier et al. (2015). The construct items for the social disconfirmation were adapted from the study of Ayyagari et al. (2011). the construct items for the hedonic disconfirmation were adapted from. The current study adapted the constructs items for anxiety and dissatisfaction from Spitzer et al. (2006) and Limayem et al. (2007). The scale construct items are adapted from the study (Maier et al., 2015). The back-translation was performed as advised in the literature, as data was collected in Mainland China, and the instrument was translated into Chinese for the convenience of the participants.

**TABLE 1:** Present construct items, factor loading, AVE, Cronbach’s Alpha, CR, *R*^2^ and *Q*^2^.

Construct Items		Loading	α	AVE	CR	*R* ^2^	*Q* ^2^
**Information disconfirmation**							
ID1	I am often distracted by the excessive amount of information available to me on social networking sites.	0.804	0.834	0.601	0.883		
ID2	I find that I am overwhelmed by the amount of information I have to process on a daily basis on social networking sites.	0.755					
ID3	There is too much information about my friends on social networking sites, so I find it a burden to handle.	0.764					
ID4	I find that only a small part of the information on social networking sites is relevant to my needs.	0.808					
ID5	I find that too much information repels me.	0.744					
**Social Disconfirmation**							
SD1	I take too much care of my friends’ wellbeing on social networking sites.	0.777	0.802	0.626	0.870		
SD2	I deal too much with my friends’ problems on social networking sites.	0.756					
SD3	My sense of being responsible for how much fun my friends have on social networking sites is too strong.	0.837					
SD4	I am too often caring for my friends on social networking sites. I pay too much attention to the posts of my friends on social networking sites.	0.793					
**Hedonic Disconfirmation**							
HD1	To access a range of apps that fulfill the purpose of pleasure	0.758	0.742	0.655	0.850		
HD2	To enjoy playing games online	0.868					
HD3	To achieve an overall sense of enjoyment	0.798					
**Anxiety**							
ANX1	Using Social networks makes me feeling nervous, anxious, or on the edge.	0.836	0.802	0.715	0.883	0.458	0.321
ANX2	After using Social networks, I am not able to stop or control worrying	0.859					
ANX3	After using Social networks, I start worrying about too many things.	0.842					
**Dissatisfaction**							
DIS1	I feel dissatisfied with my overall experience using virtual networking sites.	0.730	0.712	0.635	0.839	0.592	0.406
DIS2	I feel displeased about my overall experience using social networking sites. I feel discontented about my overall experience using virtual networking sites.	0.860					
DIS3	I feel dissatisfied with my overall experience using virtual networking sites.	0.796					
**Discontinuance Intention**							
DI1	In the future, I will use virtual networks far less than today.	0.784	0.713	0.635	0.838	0.658	0.367
DI2	I will sometimes take a short break from the virtual network and return later.	0.732					
DI3	I will deactivate my virtual network account.	0.868					

### Sample and Data Collection

The authors adopted a mixed online and offline data collection strategy to study the current study data. The mixed approach was adopted due to the COVID-19 pandemic restricting traveling and physical interaction. Before the data collection, the questionnaire was shared with 20 post-graduates for pretesting. The minor adjustments were made in the questionnaire after the recommendations from participants. The modified instrument was shared with the participants through online means. Specifically, the data collection reached the potential respondents in Anhui, Zhejiang, Jiangsu, and Xinjiang (in China). The respondents were assured that the provided information privacy will be ensured and used for research purposes only. The wjx form^1^ was used for the online questionnaire survey. All 217 online replies were complete and fit for further processing. Moreover, 217 offline questionnaires were also found correct out of 295, which makes the response rate of 74 percent approximately.

To verify the non-response bias issue, the offline and online data were compared, and results were found satisfactory. Further, Table 2 presents the demographic details of the respondents.

**TABLE 2:** The demographic profile of the respondents.

Demographic Analysis			
**Gender**	**Item**	**Total**	**%**

	Male	260	59.94
	Female	174	40.06
Age	Below 20	25	5.76
	20–25	102	23.50
	25–30	153	35.25
	30–35	105	24.19
	35 and above	49	11.29
Occupation	Students	185	42.62
	Professional	115	26.49
	Businessman	80	18.43
	Other	54	12.44
Income	Below 50 thousand	265	61.05
	51–99 thousand	121	27.88
	100–199	40	09.21
	200 +	8	01.84
Education	Graduation	183	42.16
	Masters	191	60.25
	M.phil/Ph.D	60	13.56

*n = 434.*

Table 2 summarizes the demographics of the respondents. The complete profile is given in the table below, and it shows that 230 respondents are male, making up to 52.99% of the data set, and females are 204, about 47.01% of the data sample size. The next is the respondents’ age, which shows that most respondents were young Chinese engaged with VNSs. 5.76% were below the 20 rest were between 20 and 35 years of life. The rest of the details can be found in Table 2 below:

## Results

To measure the structural model, the study implied the SEM (structural equation modeling) through the partial least square method or simply PLS. The PLS is a second-generation tool that can simultaneously run the measurement and structural model and evaluate the regression and component factors (CFA) (Hair et al., 2010). This study considers the SmartPLS 3.2.8 version for results analysis.

### Measurement Model

To ensure the construct reliability and validity, the study adopts the four necessary internal consistency, convergent validity, and discriminant validity. To do we measured Cronbach’s alpha (α), factor loadings, composite reliability (CR), and average variance extracted (AVE). The finding of factor loading is above 0.7. Similarly, all the α values are above 0.7. The value of CR is above the minimum standard of 0.5. So the results depict reasonable convergent validity. We checked discriminant validity to see whether the measure constructs are different from other constructs (Henseler et al., 2014). The results are found satisfactory and are given in Table 3. The table confirms the presence of reasonable discriminant validity.

**TABLE 3:** The discriminant validity.

	ID	SD	HD	ANX	DIS	DI
Informational disconfirmation (ID)	**0.775**					
Social disconfirmation (SD)	0.762	**0.727**				
Hedonic disconfirmation (HD)	0.631	0.621	**0.792**			
Anxiety (ANX)	0.623	0.619	0.536	**0.846**		
Dissatisfaction (DIS)	0.730	0.717	0.622	0.551	**0.797**	
Discontinuance intention (DI)	0.739	0.716	0.620	0.711	0.718	**0.797**

*HTMT ratio in bold less than 1 is acceptable criterion (Henseler et al., 2014).*

#### HTMT Criterion

This criterion is a third way to ensure discriminant validity. The standard is developed by Henseler et al. (2014). The cut of value is 0.9. The output given in the table below shows all values are below the cut-off value. Table 4 presents the HTMT criterion.

**TABLE 4:** Presents the HTMT criterion.

Items/Constructs	ANX	DI	DIS	HD	ID	SD
ANX	**0.887**					
DI	0.731	**0.855**				
DIS	0.634	0.772	**0.801**			
HD	0.762	0.852	0.775	**0.795**		
ID	0.757	0.817	0.746	0.730	**0.780**	
SD	0.760	0.700	0.782	0.729	0.708	**0.705**

*HTMT ratio in bold less than 1 is acceptable criterion (Henseler et al., 2014).*

#### Collinearity Statistics

Collinearity posits that a predictor variable can predict other variables in multiple regression models due to the correlation. Collinearity can be measured through the Variance Inflation Factors (VIF). The VIF value can range between 3.3 and 10 (Schlittgen et al., 2016). Table 5 presents the values of VIF, which all are observed to be less than 3.3. So this study has no issue of multicollinearity.

**TABLE 5:** Presents the values of VIF.

S. No.	Items	VIF	S.No.	Items	VIF
1	ANX1	1.746	**13**	ID1	1.820
2	ANX2	1.866	**14**	ID2	1.883
3	ANX3	1.612	**15**	ID3	1.912
4	DI1	**1.333**	**16**	ID4	**2.069**
5	DI2	1.412	**17**	ID5	1.772
6	DI3	1.662	**18**	SD1	1.506
7	DIS1	1.319	**19**	SD2	1.617
8	DIS2	1.630	**20**	SD3	1.822
9	DIS3	1.413	**21**	SD4	1.634
10	HD1	1.445			
11	HD2	1.503			
12	HD3	1.478			

*The minimum and maximum values are highlighted and underlined.*

### Structural Model

**FIGURE 2:**
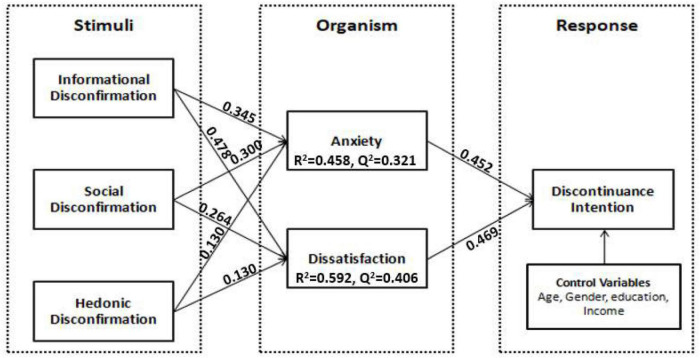
Presents the path coefficients of the structural model.

This technique shows the extent of paths between constructs and their observed effect. The current study theoretical framework is given in Figure 2, and a two-step model was used while using SEM through SmartPLS. SEM is measured through Collinearity and paths significance co-efficient.

#### Path Analysis of Structural Model

The path coefficient is measured through the SEM implied *via* SmartPLS. The path significance is measured through the bootstrapping method. Bootstrapping measures the R-square values, along with the path analysis. SmartPLS can predict up to 5,000 sample sizes. The confidential interval of 95% significance or the *t*-value > 1.96 with a two-tailed test (Schlittgen et al., 2016). The *R*^2^ > 0.2 is considered acceptable and reliable. The *R*^2^ values for the current studies are observed greater than the cut-off value. For anxiety *R*^2^ = 0.454, Dissatisfaction *R*^2^ = 0.658 and for discontinuance intention *R*^2^ = 0.654. All three *R*^2^ values are above the cut-off value of 0.2. these results further ensure the reliability of the data set. Figure 2 presents the *R*^2^ values of the present study.

The results show that the information disconfirmation positively contributes to the feeling of anxiety (β = 0.345, *p* < 0.05), informational disconfirmation makes a 34.5% contribution to the anxiety. Hence the H1a is supported. Similarly, informational disconfirmation contributes positively to service dissatisfaction (β = 0.478, *p* < 0.05), informational disconfirmation makes 47.8% contribution to service dissatisfaction. So, H1b is also supported. The second environmental perspective is social disconfirmation. Social disconfirmation makes a strong positive contribution to anxiety and dissatisfaction. The social disconfirmation contribution to anxiety is (β = 0.300, *p* < 0.05) and to dissatisfaction is (β = 0.264, *p* < 0.05). Hence H2a and H2b are supported positively. Social disconfirmation makes a 30% contribution to anxiety and 26.4% to dissatisfaction, respectively. The third environmental factor is hedonic disconfirmation. Hedonic disconfirmation also follows the trend and positively contributes to both organism factors (anxiety and dissatisfaction). The hedonic disconfirmation makes (β = 0.130, *p* < 0.05) 13% contribution to the anxiety and (β = 0.130, *p* < 0.05) 13% to the dissatisfaction accordingly. So, H3a and H3b are also supported. These findings show that hedonic disconfirmation contributes the least to the organism variables, but the informational and social disconfirmation made a major and almost equivalent contribution to the organism.

Further, anxiety makes a considerable positive contribution to the user discontinuance intentions. Anxiety makes a (β = 0.452, *p* < 0.05) 45.2% contribution to the user discontinuance intentions. Whereas, dissatisfaction makes a (β = 0.469, *p* < 0.05) 46.9% contribution to the user discontinuance intentions. So, H4 and H5 are also supported. The results show an equal contribution of both anxiety and dissatisfaction to developing user discontinuance intentions. These findings are of critical importance. The statistical results depict that environmental stimulus (informational, social, and hedonic disconfirmation) make a positive and strong contribution to the organism variables (anxiety and dissatisfaction), which ultimately influences the behavioral response (discontinuance intention). The hypotheses results in tabular format are listed in Table 6.

**TABLE 6:** Path analysis.

S.No.	Hyp.	Relation	Sample Mean (M)	Standard deviation (STDEV)	*T*-Test (|O/STDEV|)	*P*-Value	Outcome
1	H1a	ID ->ANX	0.345	0.053	6.508	0.000	Supported
2	H1b	ID ->DIS	0.478	0.056	8.485	0.000	Supported
3	H2a	SD ->ANX	0.300	0.046	6.522	0.000	Supported
4	H2b	SD ->DIS	0.264	0.052	5.086	0.000	Supported
5	H3a	HD ->ANX	0.130	0.038	3.404	0.001	Supported
6	H3b	HD ->DIS	0.130	0.043	2.985	0.003	Supported
7	H4	ANX ->DI	0.452	0.059	7.714	0.000	Supported
8	H5	DIS ->DI	0.469	0.058	8.082	0.000	Supported

*ANX, anxiety; DI, discontinuance intention; DIS, dissatisfaction; HD, hedonic disconfirmation; ID, informational disconfirmation; SD, social disconfirmation; Hyp, hypothesis.*

#### Blindfolding

A blindfolding procedure was used to evaluate the relevance of exogenous variables and the performance of the prescribed structure. This procedure is just a simple reuse of the procedure described by Mikalef et al. (2017). The method is a blend of function fitting and cross-validation. Further, this technique examines each construct’s predictive relevance by evaluating the changes in the criterion estimates (*Q*^2^) (Hair et al., 2012, 2017) consider *Q*^2^ > 0 is deemed to be of predictive relevance. The results of Stone-Geisser’s blindfolding technique (*Q*^2^) show that user anxiety is (*Q*^2^ = 0.321), dissatisfaction is (*Q*^2^ = 0.367), and discontinuance intention (*Q*2 = 0.406). All the variables have an acceptable level of predictive relevance, as all values are above the cut-off value.

### Mediation Results

This study also considers the mediation of the organism between the environmental stimuli and Behavioral response. The mediation stage presents the six hypotheses (H1c, H1d, H2c, H2d, H3c, and H3d). Table 7 shows the mediation results of these hypotheses.

**TABLE 7:** Mediation analysis.

Mediation analysis (SOR framework)							
**Hyp**	**Regression path**	**Direct effect**	**Indirect effect**	**Total effect**	**Variance accounted for (VAF)**	**Mediation results**	**Decision**

H1c	ID->ANX->DI	0.380	0.156	0.345	0.156/0.345 × 100 = 45.21%	Partial mediation	Supported
H1d	ID->DIS->DI	0.380	0.224	0.478	0.224/0.478 × 100 = 46.86%	Partial mediation	Supported
H2c	SD->ANX->DI	0.259	0.136	0.300	0.136/0.300 × 100 = 45.33%	Partial mediation	Supported
H2d	SD->DIS->DI	0.259	0.124	0.264	0.124/0.264 × 100 = 46.96%	Partial mediation	Supported
H3c	HD->ANX->DI	0.120	0.059	0.130	0.059/0.130 × 100 = 45.38%	Partial mediation	Supported
H3d	HD->DIS->DI	0.120	0.061	0.130	0.061/0.130 × 100 = 46.92%	Partial mediation	Supported

*ID, information disconfirmation; ANX, anxiety; DI, discontinuance intention; SD, social disconfirmation; HD, hedonic disconfirmation; DIS, dissatisfaction; Hyp, hypothesis.*

The variance accounted for (VAF) was measured by dividing the indirect effect with total effects and multiplied by 100 to calculate the mediation effect (Hair et al., 2013). The total impact was calculated by adding the direct and indirect path coefficients with the mediator and without the mediator. The partial, no, and complete mediation were decided on the criterion of Hair et al. (2013). The mediation effect of more than 20 and less than 80 considered partial mediation, and below 20 considered as no mediation, and above 80 considered complete mediation (Hair et al., 2013). The mediation results of this study depict partial mediation for all the mediating hypotheses. This further enhances the credibility of our results.

## Discussion and Implications

The current study investigates the consequent behavioral response of VNS discontinuance intention due to the multiple reasons presented here in detail. Specifically, the study considers the three disconfirmation constructs: informational disconfirmation, social disconfirmation, and hedonic disconfirmation. These stimuli influence the internal state of the user. The organism is the internal state of the user measure in terms of user dissatisfaction and anxiety. This negative internal state of mind leads to a negative behavioral response. The discontinuance intentions are to take a break from the VNS on a temporary or permanent basis.

The statistical findings show that the information disconfirmation positively contributes to the development of dissatisfaction and anxiety felt within the VNSs. This indicates that when the information is free fallowing and is not lemmatize, the VNSs user experiences the psychological consequences. These findings align with the literature (Liao et al., 2011) employed the EDT. They verified that regret and negative disconfirmation are implied in the website quality perspective in terms of the information quality and other views.

Social disconfirmation positively contributes to developing dissatisfaction and anxiety among the VNSs user. These findings further extend the previous outcome of Luqman et al. (2017) found that excessive socialization leads to social media exhaustion and technostress developed from the VNSs maladaptive engagement (Nawaz et al., 2018) made similar findings and considered the regret and disconfirmation of service standards as the inner mental state. This study enhances his results by introducing anxiety and dissatisfaction as a blend of the psychological and service standard failure measures.

Hedonic disconfirmation is implied when the user gets addicted to the entertainment perspective of the service, such as the online games, jokes, comments, videos, pictures, and many other things that fall in the entertainment purview. The literature review has given evidence that people do get addicted to the hedonic view of VNSs (Hsiao et al., 2016). These statistical study results are in line with these previous findings. The hedonic disconfirmation makes a positive contribution to anxiety and dissatisfaction simultaneously. The study of Luqman et al. (2017) considers the excessive engagement in the hedonic perspective as the cause of VNS exhaustion and technostress.

The dissatisfaction and anxiety were the mediating variables between the stimuli (information disconfirmation, social disconfirmation, and hedonic disconfirmation) and the behavioral response (discontinuance intention). The statistical results show that anxiety and dissatisfaction mediate the relationship between the stimuli and response. The 80/20 rule of SmartPLS is observed to verify the results (Hair et al., 2012). The mediating results show that all the six mediating paths partially mediate the relationship between the stimuli and the response, further enhancing the study’s credibility. Moreover, the organism factors mediate between the stimulus and response and these findings are in line with the study of Lin et al. (2020), his study considers the mediating role of fatigue between the environmental factors and discontinuance intention.

### Implications

This section will provide the vital theoretical and practical contribution of the study.

#### Theoretical Contribution

Like all studies, this study has also made some critical contributions to the theory and the literature, which will enhance the limits of the existing body of literature beyond the current boundaries. First, the study is a few to introduce the SOR paradigm in VNSs. The analysis introduced the SOR framework to enhance understanding the actual cause-effect relationship of the framework’s three perspectives. This enhances the framework’s applicability to diverse areas of social sciences. Second, the study considers the negative disconfirmation in three broader categories: information disconfirmation, social disconfirmation, and the hedonic disconfirmation of user expectation. As time goes on, users get addicted to the critical perspective of networking sites that lead to adverse outcomes such as work-family balance and procrastination are the most common issues. The study’s induction of user expectation disconfirmation in post-consumption behavior enhances the theoretical study contribution.

Third, this study uses the EDT to derive its three stimuli: social disconfirmation, information disconfirmation, and hedonic disconfirmation. The EDT theory is always implied in information systems studies. This study bridges consumer behavior and information system studies, so considering the EDT theory in this study extends its implementation into the consumer behavior field opening the window for the future implication of the study. Forth, the study considers the service failure measure and the psychological perspective into the technology studies that bridge the gap between the information system studies and consumer behavior studies. The prior studies were more focused on the technology perspective of the information systems. This study introduces dissatisfaction as the outcome of the service standardization failure response in post-consumption behavioral response. Second, anxiety is the psychological perspective of the framework that ensures the psychological perspective of the model. This enhances the overall contribution of the framework. In last, this theoretical study framework is derived from the EDT, SOR framework, and post-consumption consumer behavioral responses. All these three factors are enlisted here in the theoretical model that develops a robust structure for the examination and investigates the users’ behavioral responses.

#### Practical Contribution

This study has some practical contributions for VNS service providers and managers. The literature below puts forward specific useful contributions for the readers. First, the study findings prove that VNSs have a dark perspective associated with them. To gain userbase quickly through emotional services, service providers have given total freedom to the user in terms of socialization, information sharing, and hedonic involvement with VNSs. This freedom of excessive socialization needs to be restricted and more critical barriers erecting to entering one another’s social circles. This will help keep the social process in a controlled manner and reduce social stress.

Second, the free fallow of information in terms of personal generated and advertising information are the prime contributors of the informational disconfirmation in VNSs. The number of advertisements and the amount of GBs information one can view in a day or week can be restricted. This restriction cannot just be applied to one or two networks; such a measure can only be successful when used across the industry to reduce the adverse effects of excessive engagement on the overall wellbeing of the users. Third, the hedonic perspective brings many social and moral issues to society. Excessive engagement with online material has taken many precious human lives (Wasserman and Rittenour, 2018). The individual’s engagement needs restricted access with time and spans limitations.

Forth, anxiety is a severe psychological problem that can lead to many other psychological adversities. To save the consumer from such adverse effects, the service-providing executives and organizations have to look at how they can control the side effects of excessive engagement. The option of a clock showing engagement timing being online can be made part of the service. Fifth, most VNSs users report not understanding the privacy and service policy standards. The VNSs need to work on this perspective. They must make privacy and service policies more user-friendly and straightforward so that users can understand the nature of service and learn how to control themselves from adverse outcomes.

## Limitation and Future Perspective

Like other studies, the current study has limitations that could be avoided in future research attempts in information systems and human behavioral response. This section presents all limitations known to the authors for further consideration to get a better and more accurate outcome in the future. First, the data collected for this study is collected at a single point in time. This single point data is comparatively less valid than the multiple periods of data collection as each period validates the previous data collection set. Although the current study has no validity or reliability issues, future studies can imply the time series data to validate the results. Second, the study outcome is based on the 434 sample size, which is a statistically proven data sample size and meets the population’s requirement. The author suggests future studies can improve the database set, such as considering the modern technique of big data to validate the current results and see how these results react to the more extensive data set. Moreover, the data represents one country and one national culture. Future social scientists can do comparative studies that can include data sets from more than one country or one culture that will further enhance the validity of the current structure and ensure the model’s reliability.

## Data Availability Statement

The original contributions presented in the study are included in the article/supplementary material, further inquiries can be directed to the corresponding author.

## Author Contributions

All authors listed have made a substantial, direct, and intellectual contribution to the work, and approved it for publication.

## Conflict of Interest

The authors declare that the research was conducted in the absence of any commercial or financial relationships that could be construed as a potential conflict of interest.

## Publisher’s Note

All claims expressed in this article are solely those of the authors and do not necessarily represent those of their affiliated organizations, or those of the publisher, the editors and the reviewers. Any product that may be evaluated in this article, or claim that may be made by its manufacturer, is not guaranteed or endorsed by the publisher.

## Footnotes

1
www.wjx.cn
